# Prevalence of Abdominal Obesity in Children and Adolescents with Intellectual Disability in Southeastern Poland

**DOI:** 10.3390/jcm13247608

**Published:** 2024-12-13

**Authors:** Justyna Podgórska-Bednarz, Justyna Wyszyńska, Lidia Perenc, Marta Yatsula, Anna Gagat-Matuła, Artur Mazur

**Affiliations:** 1 Institute of Health Sciences, Medical College of Rzeszow University, Warzywna 1A, 35-310 Rzeszów, Poland; 2 Department of Pediatrics No. 1, Danylo Halytsky Lviv National Medical University, Pekarska 69, 79010 Lviv, Ukraine; 3 Institute of Special Education, Pedagogical University of National Education Commission in Krakow, Ingardena 4, 30-060 Kraków, Poland; 4 Institute of Medical Science, Medical College of Rzeszow University, Warzywna 1A, 35-310 Rzeszów, Poland

**Keywords:** abdominal obesity, adolescent, child, intellectual disability

## Abstract

**Background/Objectives**: The aim of the study was to determine the frequency of occurrence of a significant health problem—abdominal obesity (AO)—in children and adolescents with intellectual disability (ID) compared to children and adolescents without disabilities, examined in the period 2013–2014. **Methods**: The study group included 568 students with various ID degrees (*n* = 265 mild; *n* = 249 moderate; *n* = 54 severe) (age range 7–18 years) attending care and educational facilities. The comparison group (non-ID) was randomly selected based on the principle of matching the group (age and sex) among students without ID. Anthropometric measurements were taken: waist circumference (WC), height, and body mass. To classify WC and BMI values, percentile charts developed within the OLAF project were used. Analyses were performed using the independence chi-square test, odds ratio (95% confidence interval) and logistic regression for multivariate analysis. The level of significance was assumed as α = 0.05. **Results**: The prevalence of AO in the ID group was not statistically significant (OR = 1.31; *p* = 0.056). The risk in the study group was significantly higher in the older age category (OR = 1.88; *p* < 0.001) and increased with the level of intellectual disability, amounting (OR = 3.71; *p* < 0.001) to moderate ID and (OR = 5.62; *p* < 0.001) for profound ID, respectively. **Conclusions**: Consideration should be given to the extension of preventive and therapeutic measures to defined subgroups of children and adolescents with intellectual disabilities, who are particularly vulnerable to AO.

## 1. Introduction

The global obesity epidemic has become a very significant problem for public health worldwide. Obesity itself may not only be an aesthetic defect and, therefore, generate mental problems, but it is also a significant risk factor for the occurrence of many serious diseases in both the child and adult population. Its consequences may include diabetes, cardiovascular disease, hypertension, stroke, or some cancers [1]. However, as studies have shown to date, abdominal obesity is a stronger determinant of the occurrence of cardiovascular disease than overall obesity [2,3].

People with intellectual disabilities constitute a special subpopulation that is at greater risk of developing health problems than the general population. Its specificity is also supported by the fact that it is heterogeneous, with different degrees of intellectual disability. One of the reasons for this state is a poorer or complete lack of the ability to articulate one’s needs, including nutritional and health needs, especially in people with more severe degrees of disability [4,5]. Relatively many reports present results that indicate a statistically higher incidence of abnormalities in the nutritional status of people with intellectual disabilities and a higher incidence of overweight and obesity than in people without disabilities [6,7,8,9,10,11,12]. There are significantly fewer reports on the incidence of abdominal obesity in the analyzed group. They mainly refer to adults with ID. Based on results of previously published studies on the estimation of the frequency of AO in the pediatric population with ID that are known to the authors, these values are approximately in the range of 30–54% [13,14]. This frequency in relation to adults with ID will be 48–61%, respectively [15,16,17].

Therefore, the main aim of this study was to evaluate the incidence of AO in the ID group compared to the incidence of this disease phenomenon in the non-ID group. An additional objective of the study was to identify the basic factors that characterize the study group that significantly influence the incidence of AO.

## 2. Materials and Methods

### 2.1. Material

**Figure 1:**
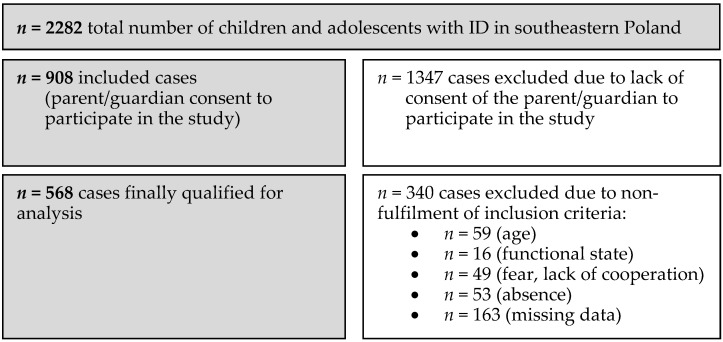
Flowchart illustrating the size of the ID group at each stage of the study.

The ID group consisted of students with ID, aged 7–18, who attended special schools in southeastern Poland during the 2013–2014 school year. Finally, 568 participants who met the inclusion criteria (age 7–18, ability to stand independently, written consent from a parent and/or child to participate in the study, correct completion and return of the questionnaire, no obvious fear of measurements being performed, cooperation during the study, presence on the day of the study) were qualified for analysis out of 2282 students attending such facilities in the defined area during that period. The detailed qualification process for the ID group is presented in Figure 1. In terms of qualification for groups depending on the degree of intellectual disability, this was assessed based on a decision on intellectual disability with a decision on the degree issued by the team for disability assessment. The team applied the assessment criteria according to ICD-10 in accordance with legal regulations based on a standardized test measuring the level of intelligence (50–69—mild degree of intellectual disability, 35–49—moderate degree of intellectual disability, 20–24—severe degree of intellectual disability) [18,19].

**Figure 2:**
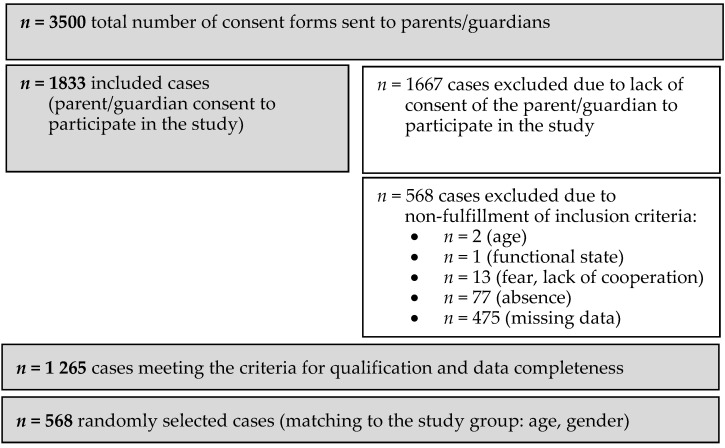
Flowchart illustrating the size of the non-ID group at each stage of the study.

The non-ID group of students without intellectual disabilities consisted of children and adolescents randomly selected from primary and secondary education institutions in a previously defined area of Poland. The following inclusion criteria were applied to this group: age 7–18, no certificate of intellectual disability, ability to stand independently, written consent from a parent and/or child to participate in the study, correct completion and return of the questionnaire, no obvious fear of measurements being performed, cooperation during the study, presence on the day of the study. In order to select the non-ID group based on matching groups (age and sex), a comparison group of 568 people was drawn using Statistica software using the random sampling function without repetitions. The detailed scheme of inclusion in the non-ID group is presented in Figure 2.

Analyses were carried out for 568 children and adolescents aged 7–18 years with intellectual disabilities (study group—ID group) and 568 children from the control group (non-ID group) without intellectual disabilities who were matched for gender and age. The characteristics of the study sample are presented in Table 1.

### 2.2. Methods

The study was part of a larger research project conducted over several years (2013–2027), aiming to verify the prevalence of overweight, obesity (including abdominal obesity), and hypertension, as well as to identify risk factors related to socioeconomic, biological, and lifestyle factors associated with these conditions. Consequently, the following methodological description is limited to the tools essential for achieving the specific objectives of this study. The study consisted of anthropometric measurements (performed in the school nurses’ offices between 10:00 a.m. and 12:00 a.m.; in accordance with the WHO recommendation of 1995: without shoes, in underwear; the average of three measurements was analyzed) and obtaining basic data relevant to the objective of the study (degree of intellectual disability based on a disability certificate and date of birth—provided with the consent of the school nurses). The age of the participant was calculated as the difference between the survey date and the date of birth, and age groups that were the midpoints of the intervals were created using the rule, e.g., age group 10 = (≥9.5 and <10.5) [20]. Then, qualifications were made for two age categories: children (7–12 years) and youth (13–18 years) [20].

### 2.3. Body Height

The measuring tool was the PORTSTAND 2010 stadiometer. Measurements were taken with an accuracy of 0.1 cm in a standing upright position.

### 2.4. Body Mass and BMI

Body mass was measured using a body composition analyzer—the Tanita BC 420 MA. On the basis of the average measurements, the BMI value was calculated (BMI = body mass [kg]/(height [m])^2^). In turn, the BMI values were referred to centile charts specially developed based on a sample representative of the Polish population of children and adolescents, created as part of the OLAF project [21,22]. The values obtained were classified according to the recommendations: underweight (<5th cc), healthy weight (≥5th and <85th cc), overweight (≥85th cc and <95th cc) [23,24].

### 2.5. WC and Abdominal Obesity

The ADE (MZ10021) waist tape measure was used to take the measurements. The waist circumference was taken at the waistline (the smallest circumference between the lower edges of the ribs arches and the upper edges of the iliac crest, at the end of free exhalation, without the tape pressing on the skin, in a standing, straight position, with the feet slightly apart, a distance of 10 cm between the feet, with the body weight evenly distributed on both lower limbs) with an accuracy of 0.1 cm [25,26]. The definition of the appearance of abdominal obesity was made based on the average WC value related to the centile charts of the OLAF project [27]. The normal distribution of fat tissue was defined for values < 95, and abdominal obesity for values ≥ 95cc [28].

### 2.6. Approval

The study was approved by the Bioethics Committee (University of Rzeszów), resolution number 9/05/2012.

### 2.7. Data Analysis

Data were analyzed using IBM SPSS STATISTICS v. 29.0. Analyses were performed using the chi-square test of independence. The odds ratio was calculated with a 95% confidence interval for the occurrence of central obesity and individual BMI categories depending on the membership of the group and the age and degree of intellectual disability. Logistic regression was used for multivariate analysis of the influence of selected factors on the appearance of central obesity. The level of significance was assumed as α = 0.05.

## 3. Results

Table 2 presents the odds ratio (OR) values for comparing the frequency of occurrence of individual BMI and WC categories among subjects from both groups. The results showed a statistically significant difference in the appearance of normal body mass and obesity. Normal body mass in subjects with intellectual disability occurred almost 40% less often than in the control group (OR = 0.61). In the study group, the odds of obesity were 2.6 times higher than in the control group (OR = 2.61). For the occurrence of underweight, overweight, and central obesity, no statistically significant differences were observed between the groups.

The appearance of central obesity was significantly more frequent among children with moderate (OR = 3.71) or severe (OR = 5.62) intellectual disability compared to mild disability. The odds of being underweight among children with moderate disabilities were on average 64% lower than among children with mild disability. The appearance of healthy body weight in children with moderate (OR = 0.45) or severe (OR = 0.29) disability compared to mild disability was 55% and 71% lower, respectively. The odds of being overweight and obese in people with moderate and severe disabilities were significantly higher—between 1.79 and 6.70 times—than in people with mild intellectual disabilities. Table 3 presents these data for the studied sample with ID.

A summary of the appearance of visceral obesity and BMI categories depending on the age of the study participants showed that central obesity occurred almost twice as often in the group of adolescents than in children (OR = 1.88). The odds of achieving a healthy body weight were 58% (OR = 0.42) lower, and the occurrence of obesity was more than three times (OR = 3.14) higher in adolescents than in children (Table 4).

Table 5 presents the odds ratio (OR) values for comparing the frequency of occurrence of individual BMI and WC categories among subjects from both groups in the age group of 7–12 years. The results showed a statistically significant difference in the occurrence of underweight and obesity, as well as overweight (overweight and obesity counted together). Underweight in subjects with intellectual disability occurred 53% less frequently than in the control group (OR = 0.47). In the study group, the odds of obesity were 2.56 times higher than in the control group (OR = 2.56). Similarly, the appearance of overweight was noted: in the study group, the odds of being overweight were 1.66 times higher than in the control group. In this age category, no statistically significant increase in the incidence of AO was observed (non-ID group =18.3% vs. ID group = 19.6%).

Similar analyses were performed in the 12- to 18-year-old group of people. The results showed a statistically significant difference in the occurrence of normal body weight and obesity. Normal body weight in subjects with intellectual disabilities occurred almost 60% less often than in the control group (OR = 0.41). In the study group, the odds of obesity were 2.76 times higher than in the control group (OR = 2.76). Significant differences were also observed for excessive body weight—the odds of obesity or overweight for subjects in the control group were 2.18 times higher than in the control group (OR = 2.18). In the older age group, a significantly higher incidence of AO was found compared to the control group (OR = 1.56). The results are presented in Table 6.

Table 7 presents the results of the multivariate stepwise logistic regression analysis. It was shown that in people with mild intellectual disability, the occurrence of central obesity depended on obesity and the interaction between overweight and the age of the subjects and the sex and age. The appearance of central obesity in people with moderate intellectual disability depended on the occurrence of overweight and obesity. Analogous results were obtained in people with severe disabilities.

## 4. Discussion

In the case of analysis of the results of our study, including the entire examined sample, there was no significantly more frequent occurrence of AO in the ID group compared to people without disabilities, such that a relationship was demonstrated in the case of general obesity—16.9% vs. 7.2%, respectively. However, when divided into age groups, a significantly more frequent occurrence of AO was demonstrated for the older age category with ID compared to the non-ID group (31.5% vs. 22.8%). Analyzing individual subgroups according to the degree of disability, it was shown that this frequency increased with the degree of ID, which was 12.5% for the mild ID group, 34.5% for the moderate ID group, and 44.4% for the severe ID group. Furthermore, the risk of the type of obesity analyzed was almost twice as high for the older age group of 13–18 (31.5% vs. 19.6%), and a similar relationship, although slightly more pronounced, was found for general obesity (25.1% vs. 9.6%). The factor that significantly influenced the frequency of AO in the entire analyzed group was the occurrence of general obesity.

Furthermore, Barria MC et al. in a study on nutritional status and cardiometabolic risk conducted in Chile in children aged 6–21 years of age from urban special education schools, found that 53.8% had a relative risk of abdominal obesity. Moreover, the results of their study indicated that children with ID were characterized by statistically higher values in weight, BMI, waist, and hip circumference [14]. Analyzing this disease phenomenon among adults with ID, Gawlik et al. showed that AO occurs more frequently in females. For the study on a sample of 27 adults with moderate ID, the percentages were 75% of women and 47% of men, respectively. This regularity also referred to general obesity [16,29], which we did not find in the case of studies in the developing population, where age was a factor significantly differentiating the occurrence of AO.

Alarming data on the prevalence of AO among people with intellectual disabilities are presented by the results of a 2020 meta-analysis by Vancampfort et al. According to researchers, AO affects more than 50% of adults with ID (52%; Cl = 42.0–61.9%) [17].

In turn, based on the results of the study by Hoey et al. conducted in a group of 135 people with ID aged 16–64 years, no correlation was found between WC and BMI, while a correlation was found between WC and degree of ID [7]. In our own study, conducted in a group aged 7–18 years, a significant relationship was found between AO and general obesity, as well as AO and the degree of intellectual disability. However, it should be remembered that the study included children and adolescents with mild to severe intellectual disability, while participants with profound intellectual disability were excluded from the study due to the cooccurrence of motor disability.

In Poland, there is relatively much talk about inequalities in the education of people with ID, but the problem of inequalities in health is somehow overlooked. One of the visible needs is to carry out screening and interventions aimed at reducing the incidence of obesity, including abdominal obesity, which is a very significant risk factor for cardiovascular and metabolic diseases [30,31]. Studies indicate that adults with ID and AO may be up to ten times more likely to develop hypertriglyceridemia, low HDL (high-density lipoprotein), hypertension, and hyperinsulinemia, compared to those without AO [32]. In connection with the fact that, as the results of studies indicate, this problem also concerns children and adolescents with ID, such actions should be introduced as early as possible and, due to the specificity of the group, cover not only individuals with ID but also their families and caregivers. Such early and broad-based prevention would certainly bring measurable results in the form of reducing the incidence and, consequently, the health consequences of abdominal obesity both in childhood and later in adulthood. However, the literature on the subject highlights the fact that in the case of the analyzed group, the quality and number of well-designed studies are still too low to unequivocally assess the potential of individual interventions that could optimally help ID individuals normalize body weight and AO [32].

## 5. Conclusions

Children and adolescents with intellectual disabilities may constitute a group that is more susceptible to the development of abdominal obesity compared to their peers without disabilities. This statement cannot be generalized in the case of our own research to the entire study group, but when considering this exposure, it should be emphasized that the frequency depends on the degree of disability—a higher degree of disability is associated with a higher risk of AO.

Other non-modifiable factors that influenced the increased incidence of AO in the ID group were the older age category and the male sex. The last factor considered in the modifiable factor group, that is, overweight and obesity (excessive body mass), also significantly influenced the increase in the incidence of AO in the study group. Of course, these reports already make us wonder about the need to implement health programs aimed at reducing the frequency of this health problem in the analyzed group, and thus reducing the frequency of its consequences, already in the population of children and adolescents with intellectual disabilities.

At this stage of the analysis, the identified subgroups underscore the importance of implementing one of the best practices in public health: enhanced surveillance. This approach, which includes regular, cyclical screening for abdominal obesity, can increase awareness of the problem’s scale and facilitate the planning of measures to prevent further increases in AO rates. In addition, it provides an important basis for the development and implementation of effective preventive and therapeutic interventions.

Recognizing abdominal obesity and identifying the groups particularly susceptible to its occurrence is a crucial first step. However, it must be followed by a detailed analysis of the complex network of factors that contribute to its development. Future analyses should prioritize examining factors such as physical activity levels, dietary habits, and the presence of comorbidities, all of which significantly influence the prevalence of this condition among children and adolescents with intellectual disabilities. The implementation of such measures will, in turn, enable the identification of actual needs, paving the way for the creation of tailor-made health programs designed to address specific challenges.

Our findings, combined with the lack of existing literature, highlight the need to conduct longitudinal studies. Such research would enable the assessment of factors influencing the occurrence of abdominal obesity over time, providing insight into the dynamics and a clearer understanding of the progression of this condition within the analyzed group.

## 6. Limitations

The ID group is a representative group for one region of Poland (southeastern), although it is not representative of the entire country. The studied sample also contains a small, although representative for this region, number of children with severe ID (*n* = 54), who constituted 9.5% of the study group. Of course, there is a need for broad-based studies that would provide results representative on a national scale and allow for further detailed analyses.

Due to the extensive dataset and numerous analyses, this study focused exclusively on the frequency of the problem analyzed, without delving into detailed risk factor analyses or conclusions regarding therapeutic and preventive programs.

As mentioned above, abdominal obesity (AO) was defined using waist circumference (WC) values from the OLAF study. Comorbidities and lipid profiles were not included in the analysis.

Future research should focus on further exploring the role of comorbidities in shaping the prevalence and impact of abdominal obesity in this population.

In future research, to gain a deeper understanding of the barriers faced by children with intellectual disabilities and their families, it will be essential to complement quantitative data with qualitative insights. This could include exploring access to health education, programs aimed at increasing physical activity levels, and other necessary support needs that have been identified.

## Figures and Tables

**Table 1:** Characteristics of the sample studied.

	Non-ID Group						ID Group					
Variables	Girls (*n* = 283)		Boys (*n* = 285)		Total (*n* = 568)		Girls (*n* = 283)		Boys (*n* = 285)		Total (*n* = 568)	
	*N*	%	*N*	%	*N*	%	*n*	%	*N*	%	*N*	%
Age												
7–12 years	146	51.6	154	54.0	300	52.8	147	51.9	154	54.0	301	53.0
13–18 years	137	48.4	131	46.0	268	47.2	136	48.1	131	46.0	267	47.0
BMI												
Underweight	29	10.2	19	6.7	48	8.5	21	7.4	21	7.4	42	7.4
Healthy weight	202	71.4	208	73.0	410	72.2	165	58.3	183	64.2	348	61.3
Overweight	33	11.7	36	12.6	69	12.1	44	15.5	38	13.3	82	14.4
Obese	19	6.7	22	7.	41	7.2	53	18.7	43	15.1	96	16.9
WC												
Proper distribution of body fat	225	79.5	227	79.6	452	79.6	200	70.7	225	78.9	425	74.8
Abdominal obesity	58	20.5	58	20.4	116	20.4	83	29.3	60	21.1	143	25.2
Degree of intellectual disability												
Mild	-	-	-	-	-	-	131	46.3	134	47.0	265	46.7
Moderate	-	-	-	-	-	-	122	43.1	127	44.6	249	43.8
Severe	-	-	-	-	-	-	30	10.6	24	8.4	54	9.5

**Table 2:** Odds ratios of the occurrence of individual BMI categories and visceral obesity in the study groups.

Classification	Non-ID Group (*n* = 568)		ID Group (*n* = 568)		OR (95% CI)	*p*
	*N*	%	*N*	%		
BMI						
Underweight	48	8.5	42	7.4	0.87 (0.56; 1.33)	0.510
Healthy weight	410	72.2	348	61.3	0.61 (0.48; 0.78)	<0.001
Overweight	69	12.1	82	14.4	1.22 (0.87; 1.72)	0.256
Obese	41	7.2	96	16.9	2.61 (1.78; 3.85)	<0.001
WC						
Proper distribution of body fat	452	79.6	425	74.8		
Abdominal obesity	116	20.4	143	25.2	1.31 (0.99; 1.73)	0.056

**Table 3:** Odds ratios of the occurrence of individual BMI categories and visceral obesity among people with intellectual disabilities.

Classification	Mild		Moderate				Severe			
	*N*	*%*	*N*	*%*	OR (95% CI)	p	N	%	OR (95% CI)	*p*
BMI										
Underweight	30	11.3	11	4.4	0.36 (0.18; 0.74)	0.004	1	1.9	0.15 (0.02; 1.11)	0.059
Healthy weight	191	72.1	134	53.8	0.45 (0.31; 0.65)	<0.001	23	42.6	0.29 (0.16; 0.53)	<0.001
Overweight	27	10.2	42	16.9	1.79 (1.07; 3.00)	0.026	13	24.1	2.80 (1.33; 5.86)	0.005
Obese	17	6.4	62	24.9	4.84 (2.74; 8.55)	<0.001	17	31.5	6.70 (3.15; 14.27)	<0.001
WC										
Proper distribution of body fat	232	87.5	163	65.5			30	55.6		
Abdominal obesity	33	12.5	86	34.5	3.71 (2.37; 5.81)	<0.001	24	44.4	5.62 (2.94; 10.76)	<0.001

**Table 4:** Odds ratios of the appearance of individual BMI categories and visceral obesity depending on age in subjects with intellectual disability.

Classification	7–12 Years (*n* = 301)		13–18 Years (*n* = 267)		OR (95% CI)	*p*
	*N*	%	*N*	%		
BMI						
Underweight	17	5.6	25	9.4	1.73 (0.91; 3.27)	0.091
Healthy weight	213	70.8	135	50.6	0.42 (0.30; 0.60)	<0.001
Overweight	42	14.0	40	15.0	1.09 (0.68; 1.74)	0.728
Obese	29	9.6	67	25.1	3.14 (1.96; 5.04)	<0.001
WC						
Proper distribution of body fat	242	80.4	183	68.5		
Abdominal obesity	59	19.6	84	31.5	1.88 (1.28; 2.77)	0.001

**Table 5:** Odds ratios of the occurrence of individual BMI categories and visceral obesity in subjects with intellectual disability aged 7–12 years.

Classification	Non-ID group (*n* = 300)		ID group (*n* = 301)		OR (95% CI)	*p*
	*N*	%	*N*	%		
BMI						
Underweight	34	11.3	17	5.6	0.47 (0.26; 0.86)	0.012
Healthy weight	219	73.0	213	70.8	0.89 (0.63; 1.23)	0.542
Overweight	35	11.7	42	14.0	1.23 (0.76; 1.98)	0.402
Obese	12	4.0	29	9.96	2.56 (1.28; 5.12)	0.006
Excessive body weight	47	15.7	71	23.6	1.66 (1.10; 2.50)	0.015
WC						
Proper distribution of body fat	245	81.7	242	80.4		
Abdominal obesity	55	18.3	59	19.6	1.09 (0.72; 1.63)	0.692

**Table 6:** Odds ratios of the occurrence of individual BMI categories and visceral obesity in subjects with intellectual disability aged 13–18 years.

Classification	Non-ID Group (*n* = 568)		ID Group (*n* = 568)		OR (95% CI)	*p*
	*N*	%	*N*	%		
BMI						
Underweight	14	5.2	25	9.4	1.87 (0.95; 3.69)	0.066
Healthy weight	191	71.3	135	50.6	0.41 (0.29; 0.59)	<0.001
Overweight	34	12.7	40	15.0	1.21 (0.74; 1.98)	0.442
Obese	29	10.8	67	25.1	2.76 (1.72; 4.44)	<0.001
Excessive body weight	63	23.5	107	40.1	2.18 (1.50; 3.16)	<0.001
WC						
Proper distribution of body fat	207	77.2	183	68.5		
Abdominal obesity	61	22.8	84	31.5	1.56 (1.06; 2.29)	0.024

**Table 7:** Factors influencing the occurrence of central obesity among people with intellectual disabilities—multivariate stepwise logistic regression analysis.

								95% CI for OR		
Step	Variables	B	SE	Wald	Df	*p*	OR	LL	UL	R^2^_Nagelkerkego_
Mild										
Step 1	Obesity	4.56	0.79	33.22	1	<0.001	95.83	20.31	452.16	0.371
	(Constant)	−2.55	0.25	108.36	1	<0.001	0.08			
Step 2	Obesity	5.76	0.87	43.93	1	<0.001	315.90	57.60	1732.61	0.629
	Overweight × Age	2.19	0.34	41.80	1	<0.001	8.93	4.60	17.34	
	(Constant)	−3.74	0.43	74.66	1	<0.001	0.02			
Step 3	Obesity	6.63	1.08	37.57	1	<0.001	753.65	90.60	6269.39	0.662
	Sex × Age	−0.89	0.36	6.19	1	0.013	0.41	0.20	0.83	
	Overweight × Age	2.71	0.45	36.66	1	<0.001	15.04	6.26	36.18	
	(Constant)	−2.27	0.67	11.34	1	<0.001	0.10			
Moderate										
Step 1	Obesity	4.76	0.63	57.53	1	<0.001	116.54	34.08	398.56	0.602
	(Constant)	−1.78	0.21	73.14	1	<0.001	0.17			
Step 2	Overweight	3.75	0.59	39.89	1	<0.001	42.67	13.31	136.78	0.768
	Obesity	6.54	0.78	70.45	1	<0.001	693.25	150.49	3193.50	
	(Constant)	−3.56	0.51	49.36	1	<0.001	0.03			
Severe										
Step 1	Obesity	2.53	0.74	11.85	1	<0.001	12.60	2.98	53.32	0.328
	(Constant)	−0.99	0.37	7.20	1	0.007	0.37			
Step 2	Overweight	2.87	0.93	9.45	1	0.002	17.60	2.83	109.56	0.531
	Obesity	3.94	0.98	16.32	1	<0.001	51.33	7.60	346.85	
	(Constant)	−2.40	0.74	10.54	1	0.001	0.09			

## Data Availability

Data is unavailable due to privacy reasons.
